# First-principle simulations of electronic structure in semicrystalline polyethylene

**DOI:** 10.1063/1.4983650

**Published:** 2017-05-22

**Authors:** A. Moyassari, M. Unge, M. S. Hedenqvist, U. W. Gedde, F. Nilsson

**Affiliations:** 1 School of Chemical Science and Engineering, Fibre and Polymer Technology, KTH Royal Institute of Technology, SE–100 44 Stockholm, Sweden; 2 ABB Corporate Research, SE–721 78 Västerås, Sweden

## Abstract

In order to increase our fundamental knowledge about high-voltage cable insulation materials, realistic polyethylene (PE) structures, generated with a novel molecular modeling strategy, have been analyzed using first principle electronic structure simulations. The PE structures were constructed by first generating atomistic PE configurations with an off-lattice Monte Carlo method and then equilibrating the structures at the desired temperature and pressure using molecular dynamics simulations. Semicrystalline, fully crystalline and fully amorphous PE, in some cases including crosslinks and short-chain branches, were analyzed. The modeled PE had a structure in agreement with established experimental data. Linear-scaling density functional theory (LS-DFT) was used to examine the electronic structure (e.g., spatial distribution of molecular orbitals, bandgaps and mobility edges) on all the materials, whereas conventional DFT was used to validate the LS-DFT results on small systems. When hybrid functionals were used, the simulated bandgaps were close to the experimental values. The localization of valence and conduction band states was demonstrated. The localized states in the conduction band were primarily found in the free volume (result of gauche conformations) present in the amorphous regions. For branched and crosslinked structures, the localized electronic states closest to the valence band edge were positioned at branches and crosslinks, respectively. At 0 K, the activation energy for transport was lower for holes than for electrons. However, at room temperature, the effective activation energy was very low (∼0.1 eV) for both holes and electrons, which indicates that the mobility will be relatively high even below the mobility edges and suggests that charge carriers can be hot carriers above the mobility edges in the presence of a high electrical field.

## I. INTRODUCTION

Polyethylene (PE) is currently used as the preferred electrical insulation material in extruded high voltage cables. The transport of electrical energy over a long distance (e.g., in inter-continental grids) requires a very high voltage in order to achieve acceptable levels of energy loss. Unfortunately, the electrical and thermal stresses on the insulation materials also increase with increasing voltage. It is therefore crucial to improve the insulation materials, by optimizing the electrical conductivity,^1^ thermal conductivity, dielectric permittivity,^2^ and electrical breakdown strength.^3^ First principle simulations can provide a better fundamental understanding of the macroscopic electrical properties, which is valuable when optimizing the material. This paper concerns the modeling of realistic polyethylene systems (amorphous, crystalline, semicrystalline, branched, and crosslinked) and first principle simulations of those systems.

The rapid development of sophisticated material modeling techniques combined with the steadily decreasing cost of computer power has enabled computer simulations to become an increasingly important complement to traditional experimental techniques for investigating and optimizing the properties of polymers. Atomistic simulation studies of purely amorphous^4^ and crystalline PE^5–8^ have been published. At room temperature, PE is semicrystalline, consisting of both amorphous and crystalline regions. The semicrystalline state, which is significantly more complex to model than the isolated amorphous and crystalline states, has received some attention.^9–14^ The complexity arises from the crystal-amorphous interface (interphase) and the fact that the packing of the stems in the crystalline phase is also affected by the structure of the amorphous phase.^15^

We have previously developed an atomistic phantom-chain model for semicrystalline PE,^11,13^ based on an off-lattice Monte Carlo (MC) algorithm, where the objective was to assess the concentrations of tie chains and trapped entanglements connecting adjacent crystals. As input to the model, a number of parameters were used, such as the molar mass distribution, crystallinity, crystal thickness, branch content and distribution, and temperature. Both single-layered PE with a single crystal thickness^13^ and multi-layered PE with a crystal thickness distribution^11^ were modeled. As a consequence of the phantom-chain MC concept, which only takes short-range *intra*molecular forces into account and thus allows atoms to overlap,^16^ the model could be used to calculate the positions of millions of atoms in less than a minute on an ordinary PC. The phantom-chain character of the model is perfectly functional for assessing ensemble averages of the inter-lamellar connections, but the structures cannot be used in simulations in which atomic resolution is required, e.g., when assessing the electronic structure of polyethylene with quantum mechanical theories such as the density functional theory (DFT). The reason is the atomic overlap due to the phantom-chain character of the chains and the potential presence of unrealistic bond lengths and bond angles where the chains enter the crystal layer. The present study presents a strategy for equilibrating such MC-generated model systems with molecular dynamics (MD) simulation in a way that allows the geometries to be used for DFT and other atomistic studies.

MD simulation is a powerful tool for studying structural and dynamical phenomena of polymeric materials on an atomistic scale. With the MD simulation method, atomistic structures can be equilibrated under the desired operational conditions, i.e., a specific combination of volume, temperature, and pressure, and structural effects of mechanical stress and deformations can be examined,^17^ molecular transport behavior can be studied,^18,19^ and thermodynamic properties can be monitored.^20^

Density functional theory (DFT) methods belong to a class of quantum mechanical methods that can be used for calculating the electronic structure and derived properties of materials. An increasing application area of DFT is dielectric materials, where different electronic structure properties are typically related to dielectric phenomena, e.g., density of states (DOS),^14^ electron/hole traps,^21–23^ HOMO (highest occupied molecular orbital)/LUMO (lowest unoccupied molecular orbital) levels,^14,24^ electronic bandgap,^14,21,25^ spatial distributions of molecular orbitals,^14,21,23,26,27^ electron/hole mobility edge,^24^ excitation and binding energies,^5^ ionization energies and electron affinities.^8,23^ Conventional DFT-methods are very computer-power-intensive and scale as *N*^3^ (*N* being the number of electrons), thus allowing only a few hundred atoms to be modeled.^28^ More computationally efficient linear scaling (LS) methods, like the method implemented in ONETEP,^29^ have been developed, and these enable thousands of atoms to be modeled. In DFT simulations, PE is usually modeled as a single isolated oligomeric chain^30,31^ or as a fully amorphous or fully crystalline polymer.^25^ Only a few studies have previously examined the electronic structure of semicrystalline PE using DFT^14^ or other theoretical approaches.^9^

**FIG. 1.**
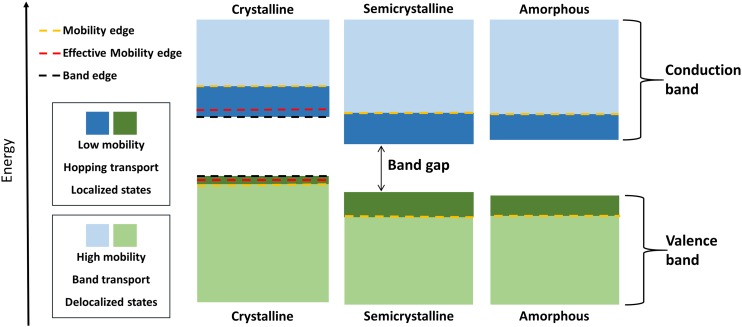
Schematic representation of the electronic structure in crystalline, semicrystalline, and amorphous PE.

This paper presents (i) a computational strategy for creating highly realistic atomistic structures of semicrystalline PE and (ii) a qualitative assessment of how the electronic structure of polyethylene (Fig. 1) is influenced by its semicrystalline structure and the presence of branches and crosslinks. This was accomplished by equilibrating MC-generated structures with MD simulation and then making calculations by applying linear scaling density functional theory (LS-DFT). The findings presented in this paper form a basis for a general interpretation of experimental data on charge transport in PE materials involving electrons and holes.

## II. METHODOLOGY AND COMPUTATIONAL DETAILS

### A. Preparation of PE model systems

The starting point was to construct the model PE using a MC method^11,13^ for the generation of the initial molecular structure and then to use energy minimization methods and MD simulation to achieve a more realistic structure. The construction strategies for the different PE model systems (crystalline, semicrystalline, amorphous, linear, branched, or crosslinked) differed slightly, depending on the type of molecular and morphological structure aimed at, and they are described in separate sections. The common simulation settings were as follows: In both the MC- and MD-simulations, all CH_2_ and CH_3_ units were modeled as united atom units. The TraPPE-UA (transferable potentials for phase equilibria, united atom version) force field,^32–34^ with the parameter values shown in Table I, was used in all energy minimizations and MD simulations on the united atom level. The TraPPE-UA force field was chosen since it can correctly account for large number of physical properties including density,^20^ radial distribution functions,^20^ conformational fluctuations,^20^ crystallization, and glass transition temperatures for PE,^20^ and offers a good balance between the computational cost and accuracy.^35^ The simulations were performed using the GROMACS 5.1.2 package,^36^ where the non-bonded Lennard-Jones interactions were calculated with a Verlet buffer cutoff scheme^37^ using a van der Waals cutoff value of 1.4 nm. To integrate Newton’s equations of motion, the leap-frog^38^ algorithm was typically used for relaxation/equilibration and production MD simulation runs. A time step of 1 fs was used in all MD runs. All atomistic structures underwent an energy minimization after adding the hydrogen atoms (prior to the ONETEP calculations) using the Materials Studio 8.0 software and PCFF (polymer consistent force field) force field. Periodic boundary conditions were applied in all three orthogonal directions. Visualization on the united-atom level was achieved using the VMD package^39^ whereas figures on the atomistic or electronic level were generated with the Materials Studio. Unless otherwise stated, the above details are valid throughout this study. The following are the details for the generation of the PE model systems.

**TABLE I.** Force field parameter values used in this work for the bonded and non-bonded interactions.^32–34^

Bond potential: $E_{bond}=\frac{1}{2}k_{b}{\left(r-r_{0}\right)}^{2}$				
${\text{CH}}_{\text{x}}-{\text{CH}}_{\text{x}}$	$k_{b}$, kJ mol^−1^ nm^−2^		$r_{0}$, nm	
	376 200.0		0.154	
Angle potential: $E_{angle}=\frac{1}{2}k_{\theta }{\left(\theta -\theta_{0}\right)}^{2}$				
${\text{CH}}_{\text{x}}-{\text{CH}}_{2}-{\text{CH}}_{\text{x}}$	$k_{\theta }$, kJ mol^−1^ rad^−2^		$\theta_{0}$, deg	
	517.2		114.0	
Torsion potential: $E_{torsion}=\sum_{0}^{n=3}C_{i}\cos^{i}\left(\varphi \right)$				
${\text{CH}}_{\text{x}}-{\text{CH}}_{2}-{\text{CH}}_{2}-{\text{CH}}_{\text{x}}$	$C_{0}$, kJ mol^−1^	$C_{1}$, kJ mol^−1^	$C_{2}$, kJ mol^−1^	$C_{3}$, kJ mol^−1^
	8.40	16.79	1.13	−26.32
Nonbonded Lennard-Jones: $E_{LJ}=4\varepsilon_{ij}\left[{\left(\sigma_{ij}/r_{ij}\right)}^{12}-{\left(\sigma_{ij}/r_{ij}\right)}^{6}\right]$; $\varepsilon_{ij}={\left(\varepsilon_{i}\varepsilon_{j}\right)}^{1/2}$; $\sigma_{ij}=\left(1/2\right)\left(\sigma_{i}+\sigma_{j}\right)$				
	$\varepsilon_{i}$, kJ mol^−1^		$\sigma_{i}$, nm	
${\text{CH}}_{2}$	0.382		0.395	
${\text{CH}}_{3}$	0.814		0.375	

#### 1. Linear semicrystalline system

**FIG. 2.**
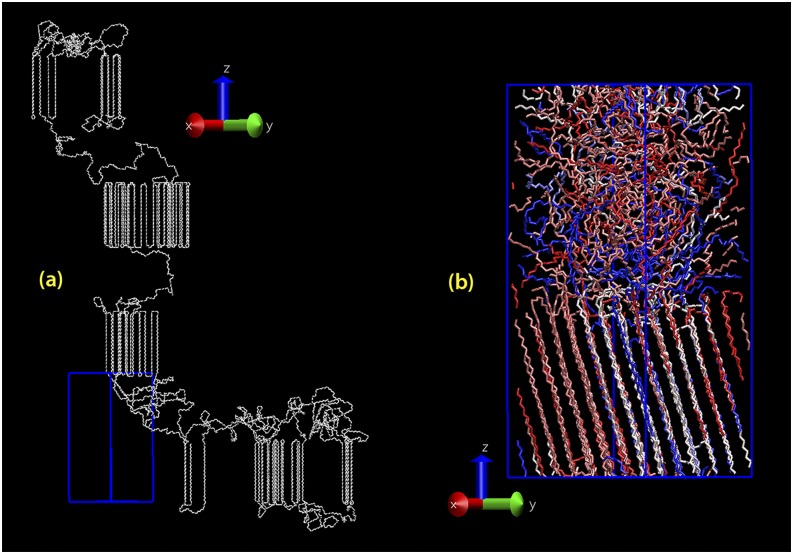
Visualization of a typical simulated semicrystalline PE. (a) Atomistic structure simulated using our MC algorithm; (b) the same structure after equilibration using MD. For clarity only the carbon atoms are shown.

The initial atomistic semicrystalline PE system, similar to the one illustrated in Fig. 2(a), was generated using the MC method developed in previous studies.^11,13^ Due to the computational cost of the succeeding electronic structure calculations with LS-DFT, relatively small PE systems consisting of about 2000 CH_x_ units were prepared. Therefore, the semicrystalline system based on linear PE consisted of only one crystal and one amorphous layer although the methodology could readily be extended to a semicrystalline multilayer system.^11^ A single linear PE chain was used to create the whole system. The dimensions of the simulation box of the initial atomistic semicrystalline system were 2.967 nm, 2.967 nm, and 4.498 nm in the **x**, **y**, and **z** directions, respectively. The **z**-axis was the normal to the crystal fold surface; the **y**-axis was the direction of crystal growth and the **x**-axis was the direction along the crystal front orthogonal to the other two directions. Since the unit cell dimensions of an orthorhombic polyethylene crystal at 298 K are *a* = 0.742 nm, *b* = 0.495 nm, and *c* = 0.255 nm,^40^ the simulation box can be described as an orthorhombic (4 × *a*, 6 × *b*, 8 × *c*) polyethylene crystal vertically aligned with a 2.462 nm thick amorphous layer.

Once the initial MC simulation was completed, an in-house code was applied in order to identify all the atoms in the crystalline layer. The atoms located in the crystal were kept frozen during the subsequent energy minimization step. The latter was thus only concerned with the amorphous subsystem by applying a steepest descent algorithm with 5000 steps. As a consequence of the geometrically constrained energy minimization, insufficiently realistic chain segments were more readily recognized as they were stretched to maintain the backbone integrity. Another code was used to identify greatly widened bond angles. These bonds were cut and hydrogen atoms were added to the new chain terminals. In this way, the energetically worst segments were replaced by chain-ends, resulting in a system with 8 chains. The new structure was energy minimized once again, using the same algorithm but with 2000 steps and no frozen atom, to become ready for MD simulations.

**FIG. 3.**
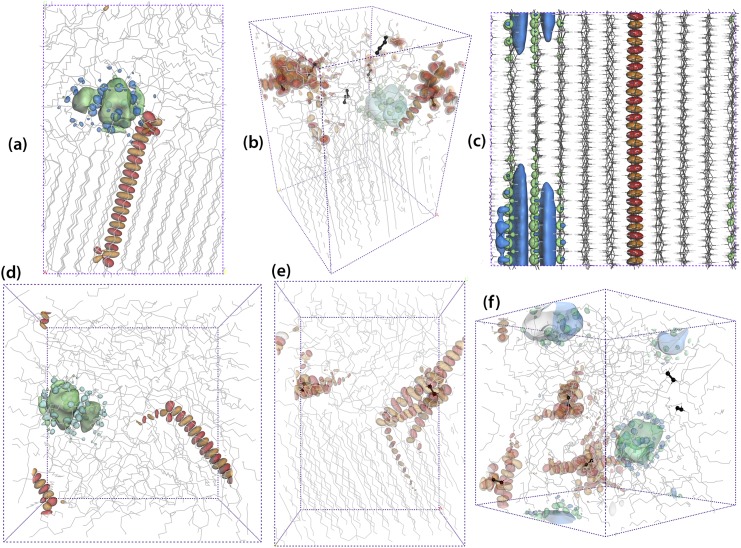
Polyethylenes after MD equilibration. HOMO/LUMO and their neighboring orbitals are illustrated with red-orange/blue-green colors. Crosslinks and branches are bolded in black. (a) Linear semicrystalline PE, (b) branched semicrystalline PE, (c) linear crystalline PE, (d) linear amorphous PE, (e) crosslinked semicrystalline PE, and (f) crosslinked amorphous PE.

Next, equilibration MD simulations, each lasting 20 ns, first on the canonical (*NVT*) ensemble and second on the isothermal-isobaric (*NPT*) ensemble, were performed. In order to keep the temperature fixed at 298 K, the v-rescale^41^ and the Nose-Hoover^42^ thermostats were used, respectively, both with a relaxation time (*τ*_T_) of 2.0 ps. Pressure was maintained at the atmospheric value (0.101 MPa) using the Berendsen^43^ barostat with a relaxation time (*τ*_P_) of 2.0 ps. For the succeeding *NPT* production run of 100 ns, Nose-Hoover (*τ*_T_ = 2 ps) and Parrinello-Rahman^44^ (*τ*_P_ = 5 ps) were used as thermostat and barostat, respectively. Fig. 3(a) shows the structure obtained after equilibration using MD simulation.

#### 2. Other polyethylene systems

In addition to the semicrystalline system based on linear PE, several other systems, as displayed in Fig. 3, were prepared: a semicrystalline system based on short-chain branched PE, a fully crystalline system based on linear PE, a fully amorphous system based on linear PE, a semicrystalline system based on crosslinked PE, and a fully amorphous system based on crosslinked PE. The mass crystallinities of all the semicrystalline systems were 50%.

The short-chain branched PE (Fig. 3(b)) was obtained by manually adding 6 butyl branches to a fully equilibrated semicrystalline system. The branches were added to the segments in the amorphous layer to ensure the exclusion of bulky branches from crystalline regions.^45^ Since the total number of backbone carbons (CH_x_) was 1688, the branch content of the model structure was 3.6 butyl groups/1000 CH_x_. After the addition of branches, the system was energy minimized again with 100 steps using the steepest descent algorithm.

The fully crystalline system (Fig. 3(c)), which consisted of 70 all-trans PE chains with both ends connected through periodic boundary conditions, was initially generated using an in-house code, relaxed with *NVT* (first, equilibration time = 20 ns) and *NPT* (secondly, equilibration time = 20 ns) MD simulations and finally equilibrated with an *NPT* MD simulation at 298 K for 1 *μ*s. The Parrinello-Rahman barostat was not successful in controlling the pressure; therefore, the MTTK (Martyna-Tuckerman-Tobias-Klein) barostat^46^ was used in the final *NPT* simulation. The MTTK barostat required the velocity Verlet^47^ integration algorithm to be used instead of the leap-frog algorithm.

The amorphous system (Fig. 3(d)) contained 10 chains, each chain consisting of 192 CH_x_ units. The chains were initially packed in a cubic periodic box (*ρ* = 850 kg m^−3^) using the algorithm of the Amorphous Cell module in the Materials Studio software. The chains were grown segment-by-segment in a three-dimensional periodic box, taking into consideration interactions with already positioned atoms while continuously monitoring the single chain conformations. In this way, an amorphous polymer structure can be built with realistic conformations and a minimum number of close contacts. The system was then relaxed and equilibrated at 298 K and 0.101 MPa through consecutive *NVT* and *NPT* runs (equilibration time = 20 ns), using GROMACS with the TraPPE-UA force field. The polymer was then gradually (during 40 ns) heated to reach 600 K, held at this temperature for 100 ns to equilibrate and finally quenched rapidly (during 5 ns) to 298 K in order to obtain a fully amorphous structure at 298 K.

Crosslinked polyethylene was also built from two fully equilibrated structures: a semicrystalline and a fully amorphous version. A new in-house code for crosslinking was applied using the unwrapped (i.e., with the periodic boundary conditions removed) carbon coordinates as input data. The algorithm identified all backbone carbons (belonging to different chains) which were closer than a specified distance. The desired number of closest pairs were crosslinked. The semicrystalline and fully amorphous systems previously prepared in this study were crosslinked using this algorithm, as shown in Figs. 2(e) and 2(f). These final crosslinked structures contained 1.19 and 2.60 crosslinks/1000 CH_x_, corresponding to the average molar masses of the chains between the junction points (${\bar{M}}_{c}$) of 11.8 and 5.4 kg mol^−1^ for the semicrystalline and amorphous systems, respectively. After the addition of the crosslinks, the systems were energy minimized with 100 steps using the steepest descent algorithm.

### B. Electronic structure calculations

#### 1. Density functional theory calculations

First, the results of conventional DFT and linear scaling density functional theory (LS-DFT), as implemented in CASTEP^48^ and ONETEP,^29^ respectively, were compared. The reference materials were (i) a small amorphous cell with a density (850 kg m^−3^) similar to that of the MD equilibrated amorphous system and (ii) a crystalline polyethylene unit cell with no thermal disorder (i.e., *T* = 0 K, *a* = 0.760 nm, *b* = 0.490 nm, *c* = 0.258 nm) and finally two crystalline systems (the same as (ii)) with addition of (iii) a vinyl group and (iv) a double bond instead of a single bond. The conventional DFT calculations were performed using the PBE generalized gradient approximation (GGA) functional^49^ and PBE0,^50^ HSE03,^51^ and HSE06^52^ hybrid functionals. The van der Waal forces were included in PBE and PBE0 via the TS method developed by Tkatchenko and Sheffler.^53^ The LS-DFT calculations were performed using the PBE functional.^49^ The energy cutoffs for these conventional DFT and LS-DFT calculations were set to 680 and 1000 eV. The kernel cutoff was about 52.9 nm (1000 bohr, i.e., effectively infinite) for the LS-DFT calculation.

Electronic structures of the relaxed PE models were then calculated using LS-DFT as implemented in ONETEP^29^ using the Materials Studio software. The GGA functional PBE^49^ and the van der Waals correction by Elstner^54,55^ were employed. The energy cutoff for the basis was set to 1000 eV and the kernel cutoff radius to 2.12 nm (40 bohr). The method used in the ONETEP code provides Kohn-sham orbitals that accurately represent the occupied states. However, the calculated unoccupied states do not necessarily represent the conduction band accurately.^56,57^ In practice, some of the states closest to the band edge were close to the correct values. States with higher energy in the conduction band and the states which significantly different from valence states are described less accurately. In this regard, an efficient method for relaxing the conduction band states has been used in ONETEP.^3,58^ Since the valence states in PE are localized along the backbone carbons and the conduction states are of an interchain nature, the characteristics of the states at the band edges can be largely different.^25^ Hence, the conduction states prior to relaxation may significantly differ from the relaxed states.

#### 2. Mobility edge calculations

In non-crystalline materials, the energies of the localized and delocalized states are separated by a sharp boundary termed the mobility edge.^59–61^ The energy difference between states at these edges and the localized ground state energy (valence/conduction band edges) (Fig. 7) is referred to as the activation energy for transport.^9^ The activation energies for electrons and holes were calculated according to the method of Unge and Christen,^24^ which was developed to analyze the transition from localized to delocalized electronic states. The states were calculated at ca. 3 × 10^6^ grid-points evenly distributed over the computational domain. At each grid point, the square of the wave function, i.e., the observation probability, was calculated and compared with a minimum probability. If the observation probability of the state was higher, that grid point was included in the ensemble of grid points with high observation probability in the electronic state. The minimum probability was determined from an imagined state that was completely delocalized over all grid points. To determine when a state become delocalized, the ensemble of grid points with high observation probability was analyzed from a percolation theory perspective. If each grid point is represented by a sphere of equal size, this 3D geometry can be analyzed. However, the spheres, i.e., grid points with high observation probability, were not randomly distributed. For example, the valence states follow the polymer backbone in PE. Therefore, a percolation threshold for non-sphere geometrical objects is needed. For amorphous PE, it has been shown that it is reasonable to use percolation threshold criteria for slightly elongated, prolate ellipsoids.^62^ For electrons and holes, the critical volume fractions were chosen as 10 and 20 vol. %, respectively, as suggested by Unge based on theoretical percolation thresholds for various ellipsoids.^62^ Unique electron/hole mobility edges were then calculated (at 0 K) for each material. Due to thermal activated hopping, additional electronic states become accessible at higher temperatures. Hence, a temperature effect was included by taking into account all the electronic states within an energy range of *k*_B_*T* (*k*_B_ being the Boltzmann constant) around the considered electronic state, resulting in an effective mobility edge at elevated temperatures. Subsequently, the activation energies for transport were obtained using mobility edge (0 K) and effective mobility edge (298 K) values (Fig. 1). In the analyses of crystalline PE, including the slightly distorted structure at 298 K, a new threshold criteria had to be determined. The states, particularly the valence states, are unidimensional following just one strand. This is further discussed in the analysis of the results.

## III. RESULTS AND DISCUSSION

### A. Characteristics of the simulated PE structures

The modeled PE densities, temperatures, energies, bond angles, torsion angles, and radial distribution functions were monitored during all the simulations, in order to check the reliability and accuracy of the model systems. All the simulated PE structures were stable and showed realistic property values.

**FIG. 4.**
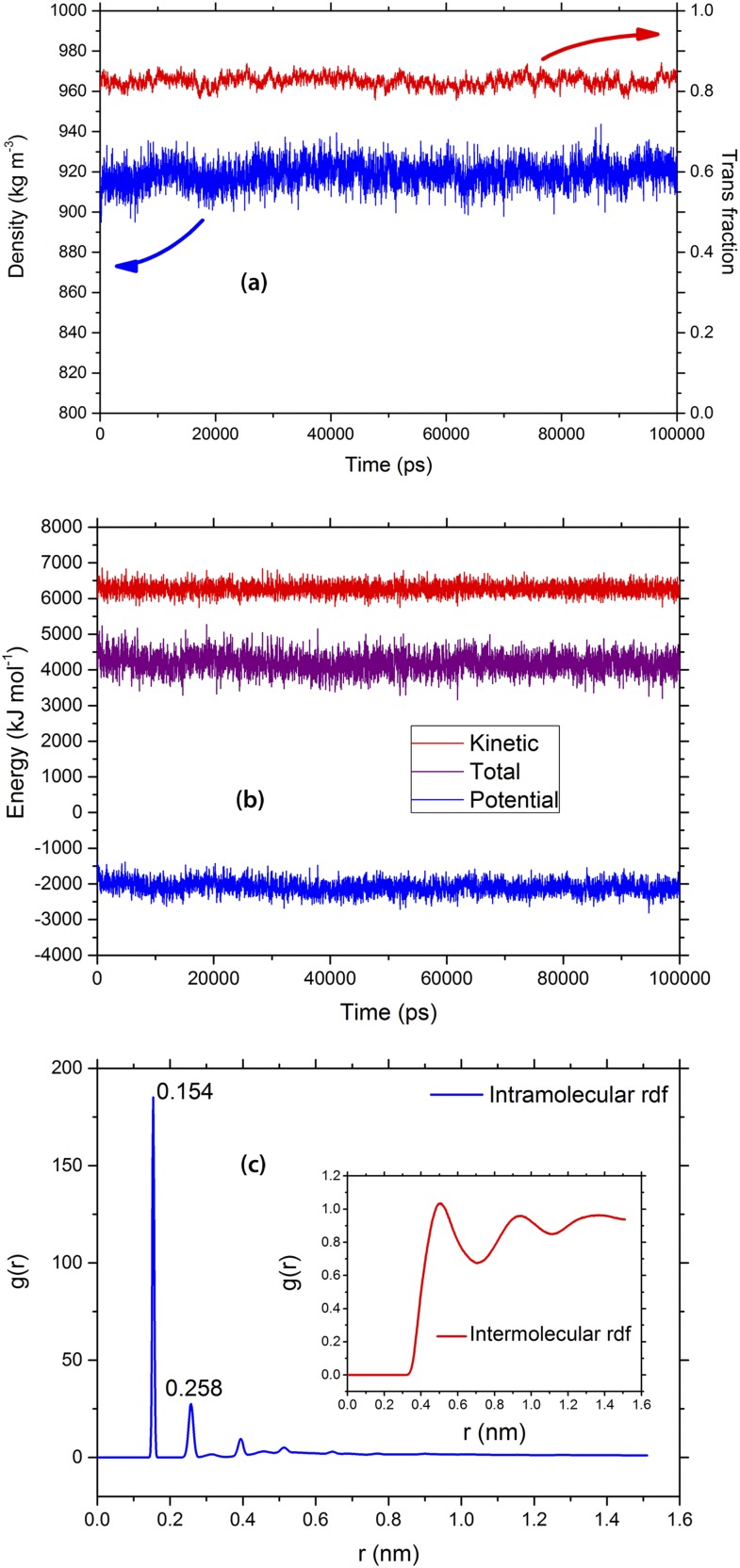
Structural properties of a MD-relaxed linear semicrystalline PE. (a) Density (blue) and trans fraction (red) versus simulation time. (b) Energy versus simulation time and (c) intra- and intermolecular radial distribution functions (RDF) versus distance (nm).

The results of the stability analysis for the equilibrated semicrystalline system based on linear PE are displayed in Fig. 4; these results were qualitatively also representative of the other modeled PE systems. Fig. 4(a) shows that the density and the fraction of torsions in the trans state are stable over the simulation time (100 ns). The density was 919 kg m^−3^ (corresponding to 50% mass crystallinity), which is a good representative of a PE used for high-voltage cable insulation material.^63^ The fraction of trans conformers, defined as the fraction of torsional angles lying between –π/3 and π/3, was 83%, which is in accordance with earlier reported simulation data.^64^ Kinetic and potential energies are shown in Fig. 4(b), showing stable energy values over the simulation time. Fig. 4(c) shows the calculated *inter*- and *intra*molecular radial distribution functions (*g*(*r*)) as a function of the distance from the atom center. The first peak (0.154 nm) of the *intra*molecular *g*(*r*) plot corresponds to the average bond length whereas the second peak (0.258 nm) corresponds to the average distance between every second carbon atom. Using these figures, the average carbon-carbon bond angle was calculated to be 113.79° for the semicrystalline system based on linear PE. Also, as in structures illustrated in Figs. 2(b) and 3(a), the crystal stems perpendicular to the lamella surface in the MC structure have tilted during the equilibration MD simulations with a tilt angle of about 25°, which is comparable with the experimentally obtained average value of 31°.^65^ These structural property values are thus comparable with the experimentally reported values for semicrystalline PE.^66^

The modeled density for purely crystalline PE (after a 300 ns equilibration *NPT* run) reached a stable ensemble average value of 1007 kg m^−3^, which is in a reasonable agreement with the reported experimental value of 1003 kg m^−3^.^67^ Lattice dimensions for the crystalline system were calculated using the first two peaks of the *inter*molecular *g*(*r*) and the second peak of the *intra*molecular *g*(*r*). The values obtained were *a* = 0.760 nm, *b* = 0.490 nm, and *c* = 0.258 nm, which are close to the experimental values at room temperature of *a* = 0.742 nm, *b* = 0.495 nm, and *c* = 0.255 nm.^40^ For the quenched amorphous structure, the density was 856 kg m^−3^, which is comparable with the reported experimental value of 852 kg m^−3^.^67^ The crosslinked amorphous systems exhibited densities similar to those of the quenched amorphous linear PE. The semicrystalline systems based on crosslinked and short-chain branched PE showed density values close to that of the semicrystalline system based on linear PE. The similarity of the structural properties of the simulated crystalline and amorphous systems with the experimentally obtained values reported in the literature supports the utility of the TraPPE-UA force field for simulating semicrystalline PE.^20,35^

### B. Electronic structure

Previously reported bandgaps of PE obtained by DFT simulation^25^ are approximately 2 eV lower than the experimentally determined bandgaps of 8.8 eV for semicrystalline PE.^68^ Recently, it was reported that hybrid functionals HSE06 and many body perturbations GW give bandgaps of the order of 8 eV for crystalline PE.^6,57^ However, for linear scaling DFT calculations of large structures, these methods are too computationally expensive or even unavailable. In order to motivate the detailed bandgap analysis of complex PE systems, although the systematic bandgap error is relatively large, the influence of different functionals was first examined on simple PE systems (Section II B 1). The conventional DFT results were compared with the corresponding LS-DFT (ONETEP) results (Table II).

**TABLE II.** Primary electronic calculation results.

	Bandgap (eV)				
		CASTEP			
			Hybrid functional		
Sample	ONETEP GGA PBE^a^	GGA PBE^b^	PBE0^b^	HSE03	HSE06
Linear amorphous (ρ = 850 kg m^−3^)	5.99	5.96	7.98	7.29	7.18
Linear crystal (ρ^c^ = 970 kg m^−3^)	6.73	6.67	8.75	8.05	7.97
Crystalline with vinyl	5.10	5.07	7.07	…	…
Crystalline with double bond	4.86	4.84	6.88	…	…

^a^ The Elstner method^54,55^ for DFT dispersion correction, as implemented in ONETEP, was used. The reported bandgap values are after conduction band relaxation.

^b^ The TS method^53^ for DFT dispersion correction, as implemented in CASTEP, was used.

^c^ The different density value compared to that of the MD relaxed pure crystalline model is due to the ideal nature of the structure at 0 K.

With the standard PBE functional, the bandgap differences between conventional DFT and LS-DFT were small (0.02–0.06 eV). This indicates that, for the purpose of this study, LS-DFT is a reasonable substitute for conventional DFT. Also, all three hybrid functionals resulted in simulated PE bandgaps much closer to the experimental value of 8.8 eV^68^ than corresponding PBE functionals. In particular, the hybrid functional PBE0-TS resulted in a bandgap of 8.75 eV for the crystalline PE system, and it is likely that HSE03/HSE06 would give similar values with van der Waals forces included. The CASTEP bandgap differences between the standard PBE and the hybrid functional PBE0-TS were in the range 2.03 ± 0.03 eV for both amorphous and crystalline system with and without chemical defects. These values are very similar. It is thus assumed that the relative bandgap differences between the different complex PE’s studied in this paper, as calculated with LS-DFT with PBE functional, are qualitatively correct even though approximately 2.03 eV should be added to achieve correct absolute values. This quantitative error could preferably be avoided in future studies by using the computationally more expensive higher level hybrid functionals.

Table III summarizes the results obtained from the LS-DFT electronic structure analysis. The bandgaps of all the samples decreased significantly (∼1 eV) after conduction band relaxation, an observation analogous with the results reported in a previous study.^62^ After conduction band relaxation, the bandgap values of linear polyethylene were placed in the following order: 5.82 eV (semicrystalline, 298 K) < 5.89 eV (amorphous, 298 K) < 6.00 eV (crystalline, 298 K) < 6.73 (crystalline, 0 K). This is in accordance with the results (5.9-6.7 eV) in other theoretical studies.^8,14^ The calculated bandgap difference between semicrystalline and crystalline systems (∼0.2 eV) is in agreement with both experimental and theoretical literature data regarding the introduction of shallow traps by conformational disorder (gauche conformations).^1,2,22^ Even though the structural change between the perfect crystal and the slightly distorted crystal due to temperature is small, the impact on the bandgap is large (0.73 eV). This can be understood from the possibility for the conduction states to relax to confined regions due to a symmetry break of the crystalline structure. The smaller bandgap of the amorphous model of PE, compared to that of the crystalline version, is in accordance with that of Unge *et al.*^14^ The bandgap of the semicrystalline model was smallest, which could be an indication of the preference for electrons to reside inside the interfacial regions of PE rather than in the crystalline part. Future analysis of a larger set of semicrystalline structures, with various compositions of tight folds and defects in the crystalline-amorphous interface,^15^ will clarify this conclusion. After the addition of short-chain branches and crosslinks, a small (∼0.05 eV) decrease in the bandgap was observed.

**TABLE III.** Electronic calculation results.

Property						
			Activation energy^b^ (eV)			
	Bandgap^a^ (eV)		Hole, using percolation threshold = 0.1		Electron, using percolation threshold = 0.2	
Sample	Initial	After conduction band relaxation	0 K	298 K	0 K	298 K
Linear semicrystalline	6.82	5.82	0.28	0.08	0.45	0.12
Branched semicrystalline	6.74	5.76	0.33	0.10	0.46	0.08
Linear crystal	6.71	6.00	∼0.47^c^	0.05	0.28	0.01
Linear amorphous	7.05	5.89	0.20	0.02	0.40	0.07
Crosslinked semicrystalline	6.81	5.75	0.31	0.09	0.45	0.10
Crosslinked amorphous	6.79	5.53	∼0.45^c^	0.28	0.42	0.06

^a^ Bandgap values are the HOMO-LUMO difference values.

^b^ Calculated for electronic structure data, obtained after conduction band relaxation, using 6th degree polynomial function fit. See Fig. 7.

^c^ Extrapolated.

**FIG. 5.**
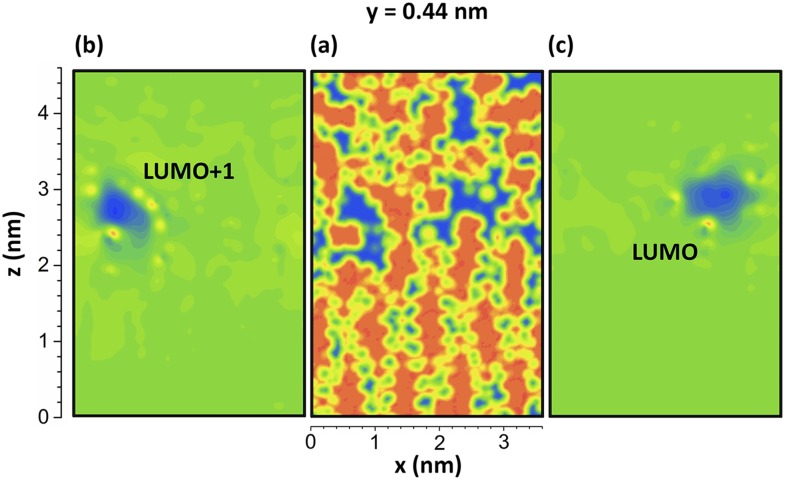
2D color maps of a plane parallel to the **x** and **z** axes at **y** = 0.44 nm, for the linear semicrystalline PE system, cutting through (a) free volume, (b) LUMO+1 orbital isosurface, and (c) LUMO orbital isosurface. The blue color shows the free volume and orbitals in the respective panels.

Fig. 3, which illustrates the carbon positions of six MD-relaxed PE systems, also includes the HOMO (red/orange) and LUMO (green/blue) as well as some higher order orbitals. The isosurface value was set to 0.01 for all orbital illustrations. For all systems, the LUMO (and above) orbitals are more diffuse in the free volumes between the chain segments (shown in Fig. 5), whereas the HOMO (and below) orbitals are mainly localized along the chain segments (Fig. 3). The observation is that HOMO levels are often localized whereas LUMO levels are more diffuse in accordance with the work of Ramprasad *et al.*^69^ In the crosslinked semicrystalline system (Fig. 3(e)), the HOMO-1 and HOMO-2 are localized around the crosslinks. The valence states (HOMO, HOMO–1 to HOMO–3) are also localized around the crosslink in the crosslinked amorphous PE system (Fig. 3(f)). A similar tendency was found for the branched system (Fig. 3(b)). In semicrystalline PE containing no branches or crosslinks (bulk semicrystalline PE), the LUMO (and neighboring levels) tends to localize at the crystalline/amorphous interface (Figs. 3(a) and 5).

**FIG. 6.**
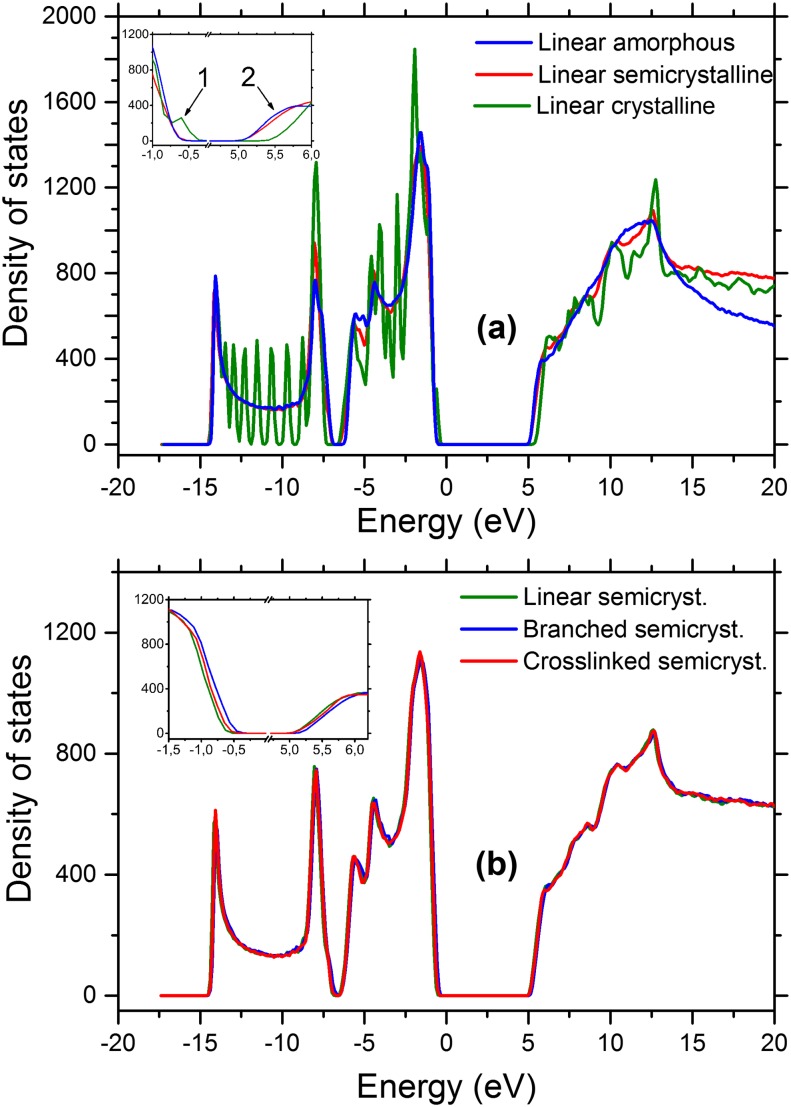
Density of states as a function of energy for (a) amorphous, crystalline, and semicrystalline PEs and (b) linear, branched, and crosslinked semicrystalline PEs. The inset arrows in (a) highlight the features distinguishing the band edge states in the periodic crystalline system from the other two models.

The density of states (DOS) is plotted as a function of energy for the model systems in Fig. 6. In Fig. 6(a), the DOS of the crystalline system has shifted horizontally so that the VBM (valence band maximum) is at 0 eV. Then, the DOS of other two systems are aligned with reference to the lower energy peaks (∼−15 to −8 eV) of the crystalline system. These reference peaks belong to the semicore states, which is a characteristic of the materials and does not change with the conformational variation. The crystal valence band edge becomes higher in energy than the other systems as shown in the inset figure with arrow number 1. These states correspond to the continuous periodic delocalized orbitals (red/orange HOMO orbital in Fig. 3(c)) which span the whole length of the crystal stems. These states only exist for fully crystalline systems as the other two systems does not have any continuous periodic chains, i.e., the lower symmetry and non-periodicity of the amorphous and semicrystalline systems are the cause of this difference in VBM’s DOS. Hence, bulk crystal properties may not be fully represented in the semicrystalline structures. Future work of examining semicrystalline structures with thicker crystalline layers up to experimental values of ca. 10-20 nm will be informative. Also, as pointed out by arrow number 2 in the inset figure, the CBM (conduction band minimum) states of the amorphous and semicrystalline system correspond to LUMO and neighbor (in energy) orbitals which are localized at the asymmetric conformational disorder (free space) regions which are at lower energies and do not exist in the fully crystalline periodic model. This is the reason for higher energy CBM of fully crystalline DOS.

The DOS values are normalized using the total number of electrons for the crystalline model. As expected, the semicrystalline DOS is an intermediate between the fully amorphous and fully crystalline systems (Fig. 6(a)) because semicrystalline PE is a combination of crystalline and amorphous regions; the only difference is the crystal/amorphous interface, which contains a high fraction of folded PE chains. The semicrystalline system has a bandgap 0.07 eV lower than that of the linear amorphous system. The tight fold at the crystal-amorphous interface includes only 8–10 CH_2_ units.^70^ These sharp turns of the polymer are unique for the crystalline-amorphous interface and yield electronic states with energy levels for the interface different from those of the bulk. In addition, the free volume at the interface is different from that of the bulk and influences how *inte*rchain states in the conduction band localize and thus their energy level. This is seen in the analysis of the calculated electronic states.

The DOS for different semicrystalline systems is plotted in Fig. 6(b). The semicore states of the linear semicrystalline system, as aligned in Fig. 6(a), was used for vertical alignment of the other semicrystalline models. The DOS values are normalized using the total number of electrons for the linear semicrystalline system. There are subtle differences, e.g., the bandgap energy values. As shown in the inset figure, the valence and conduction band edges of the branched semicrystalline system are slightly shifted towards higher energy values than those of the other semicrystalline systems; this corresponds to the new states introduced by the branching which can be considered as very shallow traps compared to the bulk states. The DOS differences for linear versus crosslinked PE were also small for amorphous PE.

### C. Mobility edge results

**FIG. 7.**
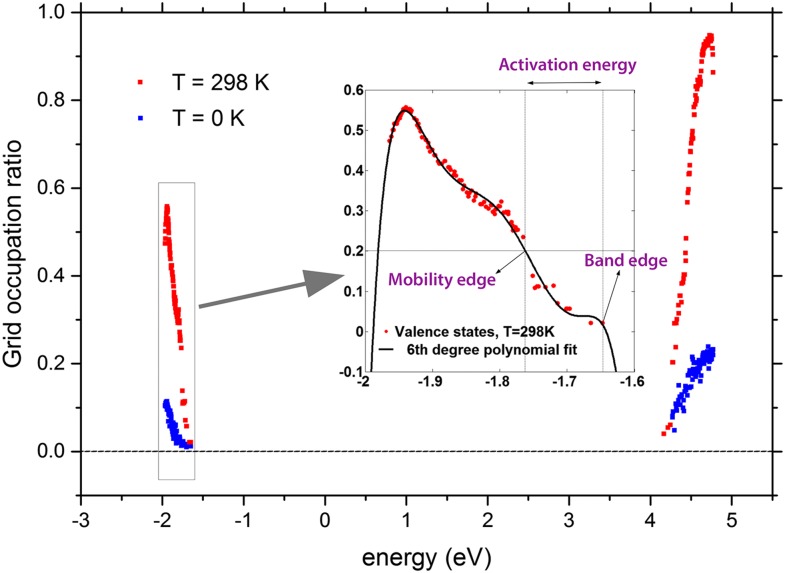
Grid occupation ratio as function of energy (eV) for the semicrystalline model geometry at 0 K and at room temperature. The inset indicated by an arrow shows an example of curve fitting using a 6th degree polynomial on the valence states at room temperature. Values of band edge, mobility edge, and activation energy are also illustrated at a percolation threshold of 0.2. Note that the activation energy calculations are performed on the raw results of each ONETEP run (without alignment). The reason is that the alignment is only a shift in energy axis and does not affect the activation energy values since activation energy values are differences between band and mobility edge values.

**FIG. 8.**
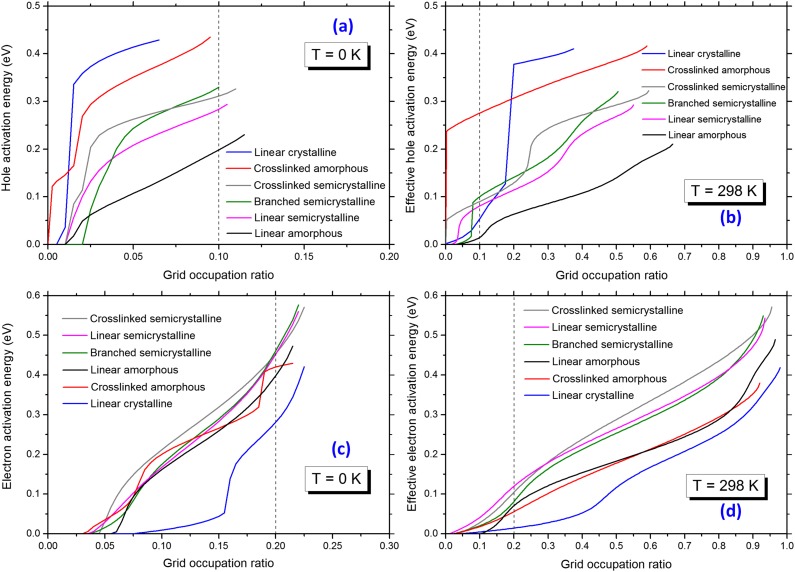
Activation energy and effective activation energy as functions of grid occupation ratio for different systems studied in this work. (a) and (c) and (b) and (d) are for calculations at 0 K and 298 K, respectively. A guideline is drawn in each subplot at a suggested percolation value.

In order to determine the mobility and effective mobility edge, the grid occupation ratio for ∼100 orbital levels closest to the band edges was calculated for the modeled systems at both 0 K and 298 K. A polynomial function was separately fitted to the data corresponding to the valence and conduction band states. As illustrated for the semicrystalline system based on linear PE in Fig. 7, the 6th degree polynomial function showed a good fit to the simulated data. The electron/hole activation energies were obtained by calculating the difference between the energy value of the fitted function at each point and the corresponding conduction/valence band edge value, i.e., the lowest/highest energy state in the conduction/valence band. The calculated activation energy values are shown as a function of the grid occupation ratio in Fig. 8. All the curves are monotonically increasing with the increasing grid occupation ratio. The percolation criteria of 0.1 (holes) and 0.2 (electrons), illustrated by the guidelines in Fig. 8, were used to calculate the activation energy value of the valence and conduction bands (explained in detail in Section II B 2). The results of this exercise are presented in Table III.

The calculated electron activation energies of the linear amorphous (0.40 eV), crystalline (0.28 eV), and semicrystalline (0.45 eV) materials are comparable with the values of 0.27, 0.28, and 0.39 (lamella phase) eV reported by Wang *et al.*^9^ The electron activation energy of 0.40 eV for the amorphous PE is double the value of 0.22 eV reported by Unge.^62^ In the previous study,^62^ the energy of the individual states, i.e., the first state above the percolation threshold, were compared to the band edge value. However, in the current study, we have used a smooth polynomial fit based on all the simulate data obtained. This difference can explain the observed discrepancy in the electron activation energies of the amorphous structures.

The hole activation energies are 0.20 eV, 0.47 eV, and 0.28 eV for amorphous, crystalline, and semicrystalline structures, respectively. The hole activation energy of 0.20 eV for the amorphous PE is less than the value of 0.3 eV reported by Unge.^62^ The high activation energy for holes in the crystalline structure may seem surprising at first glance. However, if one considers that the crystalline structure is more like a collection of unidimensional systems, the strands, rather than a true three-dimensional structure, another percolation threshold must be used when a state is delocalized along the full strand. An example can be seen in Fig. 3(c) where the HOMO is already delocalized along the strand. These states will be degenerated due to the high degree of spatial ordering in the crystalline structure. This is seen in the effective activation energy, 0.01 eV, which is lowest for the crystalline structure, indicating that there are many states close to each other in energy that are diffuse or delocalized along the strands.

Only the purely crystalline PE had an activation energy that deviated significantly from that of the other systems. All the other simulated PE systems contained an amorphous fraction, and thus this implies that the amorphous phase had a clear effect on the electron and hole activation energies of the material. This conclusion is expected because the structural disorder localizes the orbitals. However, the bandgap of the crystalline structure is the largest, so excess charge carriers will minimize their energy by residing in the amorphous phase or at the crystal-amorphous interface. Hence, any experimentally observed activation energies should be compared to the semicrystalline activation energies. The larger activation energies observed for the branched and crosslinked system are due more to the introduction of shallow traps than to a shifting of the mobility edges.

The effective activation energies are all below 0.1 eV, except for that of the amorphous system based on crosslinked PE (Table III). This indicates that the mobility of charge carriers, holes, and excess electrons can also be relatively high below the mobility edges, via a hopping conduction mechanism. However, above the mobility edge, the transport will be band-like and the charge carriers are expected to be highly mobile. In the presence of a high electric field, the charge carriers located above the mobility edge may be hot, in particular the excess electrons may initiate electron avalanches and other pre-breakdown events. As long as the charge carriers are below the mobility edge (closer to the band edge), the mobility is expected to be low, but still at a level comparable to the mobility level prevailing in the leakage (polarization) current.^71^

## IV. CONCLUSIONS

Realistic molecular structures of PE have been generated with MD-simulations on the united-atom level. The initial semicrystalline PE structures were constructed with a MC phantom-chain model and were subsequently equilibrated with MD. Densities, bond angles, and crystal lattice dimensions were close to the experimental values. Amorphous, crystalline, and semicrystalline systems based on linear, branched, and crosslinked PE were modeled. LS-DFT was applied to all the modeled systems in order to obtain information about the electronic structure of the different types of PE. The HOMO’s were localized on the chain segments. In the branched and crosslinked structures, the HOMO’s and neighboring levels were positioned around the branches and crosslinks. The conduction band states were localized in the free volume of the non-crystalline parts. Especially, the LUMOs were observed in the free volume at the interfaces between crystalline and amorphous regions. The relaxation of conduction states in the free volume and the new states in the valence band connected to the tight chain folds at the amorphous crystalline interface result in a smaller bandgap at the interface. The small decrease in the bandgap of semicrystalline (∼0.2 eV) in comparison with that of crystalline system is in accordance with the shallow trap depths reported elsewhere for the introduction of shallow traps by conformational disorder (gauche conformations). A small (0.05 eV) decrease in the bandgap was observed for the systems based on crosslinked and branched polymer chains; this shift is assigned to shallow traps associated with branches and crosslinks. Since the introduced crosslinks and short-chain branches are added with real proportions and configurations with respect to the real material used for HVDC cable insulation, we can say that these introduced states do not affect the bandgap of PE heavily. However, it is well known that the byproducts of the crosslinking process have significant effects on electrical properties of PE. Conventional DFT successfully confirmed the LS-DFT results for crystalline and amorphous PE, in accordance with the bandgap obtained experimentally. Finally, the activation energy for transport was calculated for electrons and holes, and the results obtained were comparable with the results reported by Unge^62^ and by Wang *et al.*^9^ The activation energy is lower for holes than for electrons at 0 K. At 298 K, however, both the electron and hole activation energies are very low (0.1 eV), which indicates that the mobility will be relatively high even below the mobility edges due to a hopping conduction mechanism.^71^ Above the mobility edge, the mobility is expected to be high, with the possible implication that the charge carriers will be hot.
